# Pilot Study of Cannabidiol for Treatment of Aromatase Inhibitor‐Associated Musculoskeletal Symptoms in Breast Cancer

**DOI:** 10.1002/cam4.71117

**Published:** 2025-08-01

**Authors:** Nicole M. G. Fleege, Elise A. Miller, Kelley M. Kidwell, Zeb R. Zacharias, Jon Houtman, Kelly Scheu, Kathleen Kemmer, Kevin F. Boehnke, N. Lynn Henry

**Affiliations:** ^1^ University of Michigan Medical School Ann Arbor Michigan USA; ^2^ Department of Biostatistics University of Michigan School of Public Health Ann Arbor Michigan USA; ^3^ Human Immunology Core Holden Comprehensive Cancer Center Iowa City Iowa USA

**Keywords:** aromatase inhibitor associated musculoskeletal symptoms, breast cancer, cannabidiol, cannabis, clinical trial, endocrine therapy, quality of life, supportive care

## Abstract

**Introduction:**

Aromatase inhibitor (AI) therapy reduces breast cancer recurrence risk. However, some patients stop treatment early because of AI‐associated musculoskeletal symptoms (AIMSS). AIMSS is due in part to systemic inflammation. Cannabidiol (CBD) has anti‐nociceptive and anti‐inflammatory properties, making it a potential treatment option for AIMSS.

**Methods:**

Women with stage 0–3 hormone receptor‐positive breast cancer experiencing AIMSS enrolled in this phase 2 clinical trial. Patients received CBD (Epidiolex), titrated over 4 weeks to 100 mg BID, for a total of 15 weeks. Patient‐reported outcomes were collected serially. The primary endpoint was the number of patients with at least a 2‐point reduction in worst pain from baseline to 15 weeks. Statistical analysis was completed using paired *t*‐tests and linear mixed models.

**Results:**

Of 39 eligible patients, 28 completed protocol‐directed study treatment. Eleven discontinued treatment due to toxicity (*n* = 5) or per patient preference (*n* = 6). Seventeen of 39 patients met the primary endpoint (43.6%, 95% CI [28%, 60%]). Worst pain improved 0.13 per week of treatment (*p* < 0.001) for all patients; average improvement in worst pain was 1.95 points at the end of 15 weeks. Of the 28 patients who completed the study, average reduction in worst pain was 2.36 points (95% CI [−3.22, −1.49]) between baseline and Week 15.

**Conclusion:**

Treatment with CBD was safe, tolerable, and associated with improvement in joint pain for a subset of patients. Additional studies are needed to further understand the impact of CBD on AIMSS and which patients are most likely to benefit from CBD treatment.

**Trial Registration:**

www.clinicaltrials.gov: NCT04754399

## 1 Introduction

Aromatase inhibitors (AI) are effective for reducing the risk of breast cancer recurrence in postmenopausal women diagnosed with hormone receptor‐positive disease [1]. However, up to half of patients will develop joint and muscular pain and stiffness, called aromatase inhibitor‐associated musculoskeletal symptoms (AIMSS), leading to premature discontinuation of this potentially life‐saving treatment [2, 3, 4]. The etiology of AIMSS is poorly understood but is thought to be due to the anti‐nociceptive effects of estrogen and the presence of ongoing inflammation [5]. Although there are phase III trial data to support using acupuncture, duloxetine, and exercise for treatment of AIMSS, there are also challenges limiting each strategy's widespread use [6, 7]. This highlights the need to identify additional well‐tolerated and effective interventions to improve AIMSS.

One potential intervention is cannabidiol (CBD), an exogenous cannabinoid found in *Cannabis sativa* . Preclinical studies and preliminary clinical trials suggest that CBD has both anti‐inflammatory and anti‐nociceptive properties, while lacking the psychoactive effects associated with Δ^9^‐tetrahydrocannabinol (THC) use [8, 9, 10, 11]. CBD has also shown promise for managing other pain‐related symptoms, including anxiety and insomnia [12, 13, 14, 15, 16, 17]. Given its potential impact on both pain and pain‐related symptoms, we performed this open‐label pilot study to assess CBD's clinical and mechanistic effects among individuals with AIMSS. We hypothesized that CBD would be well tolerated and result in improvements in AIMSS‐related pain.

## 2 Materials & Methods

### 2.1 Study Population

Postmenopausal women with new or worsening AIMSS while on adjuvant AI therapy for stage 0 to 3 hormone receptor‐positive breast cancer were enrolled in this single arm, single center pilot study between March 2021 and May 2023 (www.clinicaltrials.gov). AIMSS was defined as worst joint pain of at least 4 out of 10 on the Brief Pain Inventory (BPI) [18, 19] during the 7 days prior to enrollment. Participants were required to be taking standard dose AI therapy [anastrozole (Arimidex; AstraZeneca Pharmaceuticals, Wilmington, DE) 1 mg, exemestane (Aromasin; Pfizer, New York, NY) 25 mg, or letrozole (Femara; Novartis, Basel, Switzerland) 2.5 mg] orally daily for at least 3 weeks and no more than 2 years at the time of enrollment. Menopausal status was defined by standard clinical criteria or with concomitant use of luteinizing hormone‐releasing hormone (LHRH) agonist therapy. The use of cannabidiol, THC, or marijuana (oral, inhaled, or topical) within 6 weeks prior to enrollment was prohibited. Participants were ineligible if they had metastatic breast cancer, baseline total bilirubin > 1.5× upper limit of normal (ULN), AST/ALT > 3× ULN, or a history of seizures or suicide attempt. A complete list of inclusion and exclusion criteria is available in Table S1. The protocol was approved by the University of Michigan Institutional Review Board, and all participants provided written informed consent prior to enrollment.

### 2.2 Treatment Plan

Enrolled participants were treated with a Food and Drug Administration‐approved formulation of cannabidiol (Epidiolex; Jazz Pharmaceuticals, CA) that was titrated weekly by 25 mg BID per week over 4 weeks as tolerated. The initial dose was 25 mg BID (dose level 1), and the maximum dose was 100 mg BID. Participants continued the maximum tolerated dose for a total treatment duration of 15 weeks.

Participants taking analgesics for pain must have been taking a stable dose for at least 30 days prior to enrollment. They were allowed to continue this medication during study participation but were advised to remain on the same dose throughout the study. Participants were also instructed to avoid taking concomitant medications with the potential to interact with cannabidiol as described in the package insert [20].

### 2.3 Subject Assessment

During study participation, participants completed toxicity assessments by phone weekly for the first 4 weeks and then at Weeks 8, 12, and 15. Validated patient‐reported outcomes (PRO) questionnaires, including the BPI, Patient‐Reported Outcomes Measurement Information System (PROMIS)‐29 + 2 Profile v2.1 [21], Global Ratings of Change (GRC) Scale [22], Mao Expectancy of Treatment Effect (METE) [23], 2011 Fibromyalgia (FM) Survey [24], Coping Strategies Questionnaire catastrophizing scale [25], and Positive and Negative Affect Scale (PANAS) [26], were collected electronically via REDCap throughout the study period (Figure S1, Table S2) [27, 28]. Plasma samples for correlative studies were obtained at baseline, Week 8, and Week 15, and stored at 80°C until analyzed. Estradiol concentration was assessed using liquid chromatography‐tandem mass spectrometry (LC–MS/MS), utilizing a nine‐point standard curve over a concentration range of 0–200 pg/mL. Some plasma samples were acquired using the TruCulture System (Myriad RBM, Texas), collected in tubes with either lipopolysaccharide (“LPS sample”) or no lipopolysaccharide (“null sample”), to perform stimulated cytokine analysis [29]. Unstimulated and stimulated plasma samples were analyzed for interferon (IFN)α, IFNγ, interleukin (IL)‐1β, IL‐4, IL‐5, IL‐6, IL‐7, IL‐8, IL‐10, IL‐12p40, IL‐12p70, IL‐13, IL‐15, IL‐17A, IL‐17E/IL‐25, IL‐18, chemokine ligand (CCL)2, CCL3, CCL4, monocyte chemoattractant protein (MCP)‐1 and tumor necrosis factor (TNF)α using a customized 20‐plex Milliplex Human Cytokine/Chemokine/Growth Factor Panel A per the manufacturer's instructions (Millipore Sigma, Burlington, MA). Data were acquired on a Bio‐Plex 200 (Bio‐Rad, Hercules, CA).

### 2.4 Statistical Analysis

The primary endpoint of this study was the percentage of participants who achieved at least a 2‐point reduction in BPI worst pain from baseline to 15 weeks. Based on prior studies of AIMSS, we estimated that at least 70% of participants treated with cannabidiol would experience a 2‐point reduction in worst pain with 15 weeks of treatment [6]. With a sample size of 40 patients, a 1‐sided 95% confidence interval (CI) for the true proportion of participants who experienced at least a 2‐point reduction in worst pain was expected to be 51% to 81%. Participants were considered evaluable for the primary endpoint and for toxicity assessment if they met all eligibility criteria and took at least 1 dose of cannabidiol. Participants who achieved at least a 2‐point reduction in worst pain at 15 weeks were considered responders for the primary endpoint. Participants who either did not achieve a 2‐point reduction in worst pain or did not complete 15 weeks of protocol‐directed therapy were considered non‐responders for the primary endpoint.

Secondary measures included (1) the proportion of participants with at least a 2‐point reduction in BPI average pain from baseline to 15 weeks, (2) the difference from baseline at each timepoint for all other patient‐reported outcomes (sleep disturbance, fatigue, physical function, anxiety, depression, and cognitive function), and (3) the proportion of individuals with undetectable levels of estradiol at baseline and 8 weeks. Exploratory measures included the change from baseline in inflammatory biomarkers at 8 and 15 weeks, and the association between the change from baseline in biomarkers and the change in BPI worst pain score.

Baseline measures were characterized using descriptive statistics. Differences in demographics between participants with complete versus incomplete study follow‐up were evaluated using *t*‐tests and Fisher's exact tests. Exact binomial confidence intervals were constructed for proportions of responders. Comparisons of outcomes from baseline to subsequent timepoints were conducted using paired t‐tests (Week 15 BPI worst pain), sign tests (Week 15 patient‐reported outcomes), or Wilcoxon Signed‐Rank tests (cytokine and estradiol concentrations at Week 8 and Week 15). Changes in outcomes over time were evaluated using linear mixed effects models. All models for BPI outcomes included fixed effects for baseline outcome score and number of weeks (continuous), and a random intercept and slope. Models used to determine predictors of change in pain also included the baseline value of the proposed predictor as a fixed effect. Models for PROMIS outcomes included the baseline PROMIS score and number of weeks (categorical) as fixed effects, as well as a random intercept. Responders and non‐responders were compared based on baseline patient‐reported outcomes and cytokine levels (at baseline, week 8, and Week 15) using the Wilcoxon Rank‐Sum test. Spearman's correlation was calculated to identify how change in worst pain from baseline related to baseline patient‐reported outcomes and change in cytokine levels from baseline. All calculations were performed in R. Linear mixed effects models were fit using R package lme4. No adjustments for multiple comparisons were made since this was an exploratory pilot study.

## 3 Results

**FIGURE 1:**
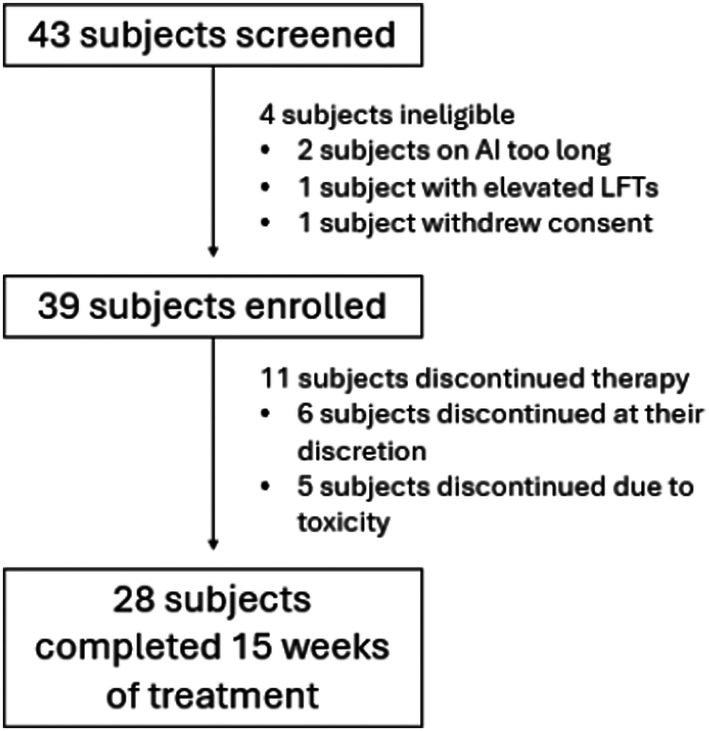
Patient flow diagram.

Between March 2021 and May 2023, 39 participants enrolled and completed baseline questionnaires and plasma collection (Figure 1). The characteristics for the 39 eligible participants are included in Table 1. Data was collected at 10 time points between baseline and Week 15 (Figure S2). Eighty percent of participants' final dose of CBD was 100 mg BID (*n* = 32, 82.1%).

**TABLE 1:** Baseline participant characteristics.

Patient characteristic	Overall (*n* = 39)	Complete (*n* = 28)	Incomplete (*n* = 11)	*p*
Age at enrollment	59.2 (10.8)	60.2 (10.5)	56.7 (11.6)	0.403
Race				
White	36 (92.3%)	25 (89.3%)	11 (100%)	1
Black	2 (5.1%)	2 (7.1%)	0 (0%)	
Asian	1 (2.6%)	1 (3.6%)	0 (0%)	
Ethnicity				
Non‐hispanic	38 (97.4%)	27 (96.4%)	11 (100%)	1
Hispanic	1 (2.6%)	1 (3.6%)	0 (0%)	
Body Mass Index	30.8 (6.57)	31.7 (6.84)	28.7 (5.54)	0.177
Prior chemotherapy				
None	26 (66.7%)	18 (72.7%)	8 (72.7%)	1
TC	10 (25.6%)	7 (25.0%)	3 (27.3%)	
ACT	2 (5.1%)	2 (7.1%)	0 (0%)	
TH	1 (2.6%)	1 (3.6%)	0 (0%)	
Surgery type				
Lumpectomy	23 (59.0%)	16 (57.1%)	7 (63.6%)	1
Mastectomy	16 (41.0%)	12 (42.9%)	4 (36.4%)	
Radiation				
Yes	29 (74.4%)	22 (78.6%)	7 (63.6%)	0.424
No	10 (25.6%)	6 (21.4%)	4 (36.4%)	
Aromatase inhibitor therapy				
Anastrozole	28 (71.8%)	21 (75.0%)	7 (63.6%)	0.285
Exemestane	8 (20.5%)	4 (14.3%)	4 (36.4%)	
Letrozole	3 (7.7%)	3 (10.7%)	0 (0%)	
Maximum dose of CBD				
100 mg	36 (92.3%)	27 (96.4%)	9 (81.8%)	0.187
75 mg	2 (5.1%)	1 (3.6%)	1 (9.1%)	
25 mg	1 (2.6%)	0 (0%)	1 (9.1%)	

*Note:* Data are provided for all participants combined and separated into those who completed all 15 weeks of study treatment (complete) and those who did not (incomplete). *p*‐values compare complete versus incomplete, using student's *t*‐test (continuous variables) or Fisher's exact test (categorical variables) where appropriate. Values listed as mean (SD) unless otherwise specified.

Abbreviations: ACT, doxorubicin, cyclophosphamide, and paclitaxel; CBD, cannabidiol; TC, docetaxel and cyclophosphamide; TH, paclitaxel and trastuzumab.

### 3.1 Baseline Patient‐Reported Outcomes

As assessed using the BPI, mean average pain at time of study enrollment was 5.18 (standard deviation (SD) 1.54), mean worst pain was 7.23 (SD 1.31), and mean pain interference score was 3.99 (SD 2.09) (Table 2). Compared to the general population, at baseline participants reported lower levels of depression (mean 45.7, SD 6.61), but higher levels of fatigue (56.5 [6.89]), sleep disturbance (54.7 [6.79]), physical function (40.7 [6.51]), and ability to participate in social activities (46.9 [7.13]). Except for PROMIS anxiety (52.8 [8.28] vs. 45.4 [6.76], *p* = 0.006), there was no difference in baseline PROs between responders and non‐responders.

**TABLE 2:** Baseline patient‐reported outcomes.

Measure	Overall (*n* = 39)	Non‐responder (*n* = 22)	Responder (*n* = 17)	*p*
BPI worst pain	7.23 (1.31)	7.27 (1.20)	7.18 (1.47)	0.92
BPI average pain	5.18 (1.54)	5.27 (1.64)	5.06 (1.43)	0.65
BPI pain interference	3.99 (2.09)	4.25 (2.19)	3.66 (1.97)	0.43
METE	13.0 (3.48)	13.1 (4.09)	12.9 (2.67)	0.86
Fibromyalgia survey criteria (Median [Range])	13.0 (1.0–26.0)	13.0 (1.0–26.0)	13.0 (4.0–24.0)	0.91
Coping Strategies Questionnaire Catastrophizing Scale	0.654 (0.720)	0.817 (0.815)	0.451 (0.539)	0.12
PANAS—positive (median [range])	31.5 (13.3–44.0)	33.0 (13.3–44.0)	30.0 (20.0–44.0)	0.59
PANAS—negative (median [range])	15.0 (10.0–31.0)	15.0 (10.0–31.0)	14.0 (10.0–21.0)	0.57
PROMIS cognitive function	51.3 (10.0)	49.1 (10.2)	53.9 (9.36)	0.09
PROMIS depression	45.7 (6.61)	46.8 (7.62)	44.4 (4.99)	0.38
PROMIS ability to participate in social activities	46.9 (7.13)	45.5 (7.37)	48.7 (6.60)	0.11
PROMIS anxiety	49.5 (8.41)	52.8 (8.28)	45.4 (6.76)	0.006
PROMIS physical function	40.7 (6.51)	39.0 (5.75)	42.7 (7.00)	0.071
PROMIS fatigue	56.5 (6.89)	56.7 (8.00)	56.4 (5.44)	0.73
PROMIS sleep disturbance	54.7 (6.79)	55.0 (6.81)	54.3 (6.96)	0.36

*Note:* Data are provided for all participants combined and separated into those who responded versus did not respond to treatment. Responders were defined as those with at least a 2‐point reduction in worst pain. Participants who did not complete the study were classified as nonresponders. *p*‐values were calculated using the Wilcoxon Rank‐Sum test. Values listed as mean (SD) unless otherwise specified.

Abbreviations: BPI, brief pain inventory; METE, mao expectancy of treatment effect; PANAS, positive and negative affect scale; PROMIS, patient‐reported outcomes measurement information system.

### 3.2 Safety and Tolerability

Safety was assessed in all 39 participants who received at least 1 dose of study medication. Adverse events were similar to those reported in prior trials of cannabidiol [30]. A total of 40 adverse events were reported (Table 3) in 18 patients (46.2%), all of which were grade 1 or 2. Eleven participants discontinued therapy during the study period, 5 because of adverse events and 6 due to lack of perceived benefit of study treatment (Table S4).

**TABLE 3:** Adverse events.

Toxicity	Any grade	Grade 1	Grade 2
Any adverse event	40 (100%)	34 (85%)	6 (15%)
Nausea	4 (10%)	4 (100%)	0 (0%)
Headache	3 (7.5%)	3 (7.5%)	0 (0%)
Stomach pain	3 (7.5%)	3 (7.5%)	0 (0%)
Diarrhea	2 (5%)	1 (2.5%)	1 (2.5%)
Elevated creatinine	2 (5%)	2 (5%)	0 (0%)
Arthralgia	2 (5%)	2 (5%)	0 (0%)
Neuropathy	2 (5%)	1 (2.5%)	1 (2.5%)
Fatigue	2 (5%)	2 (5%)	0 (0%)
Rash	2 (5%)	1 (2.5%)	1 (2.5%)

*Note:* Events included if they occurred in 5% or more of participants. No grade 3 or 4 adverse events were noted during the study. Five patients discontinued study participation due to the following adverse events: grade 1 fatigue, grade 2 neuropathy, grade 2 rash, grade 2 agitation, and for 1 patient, both grade 1 dizziness and grade 1 stomach pain. Number of events reported (percent).

Plasma estradiol concentrations were available for 38 of 39 participants. There was no significant change in estradiol concentration from baseline to Week 8. The median estradiol concentration at baseline was 0.600 pg/mL (range 0.300–1.90) and 0.600 pg/mL at Week 8 (0.300–19.0). One participant had an estradiol level of 19.0 pg/mL at Week 8, but all other patients had an estradiol value < 3.0 pg/mL.

### 3.3 Reduction of Pain With CBD

**FIGURE 2:**
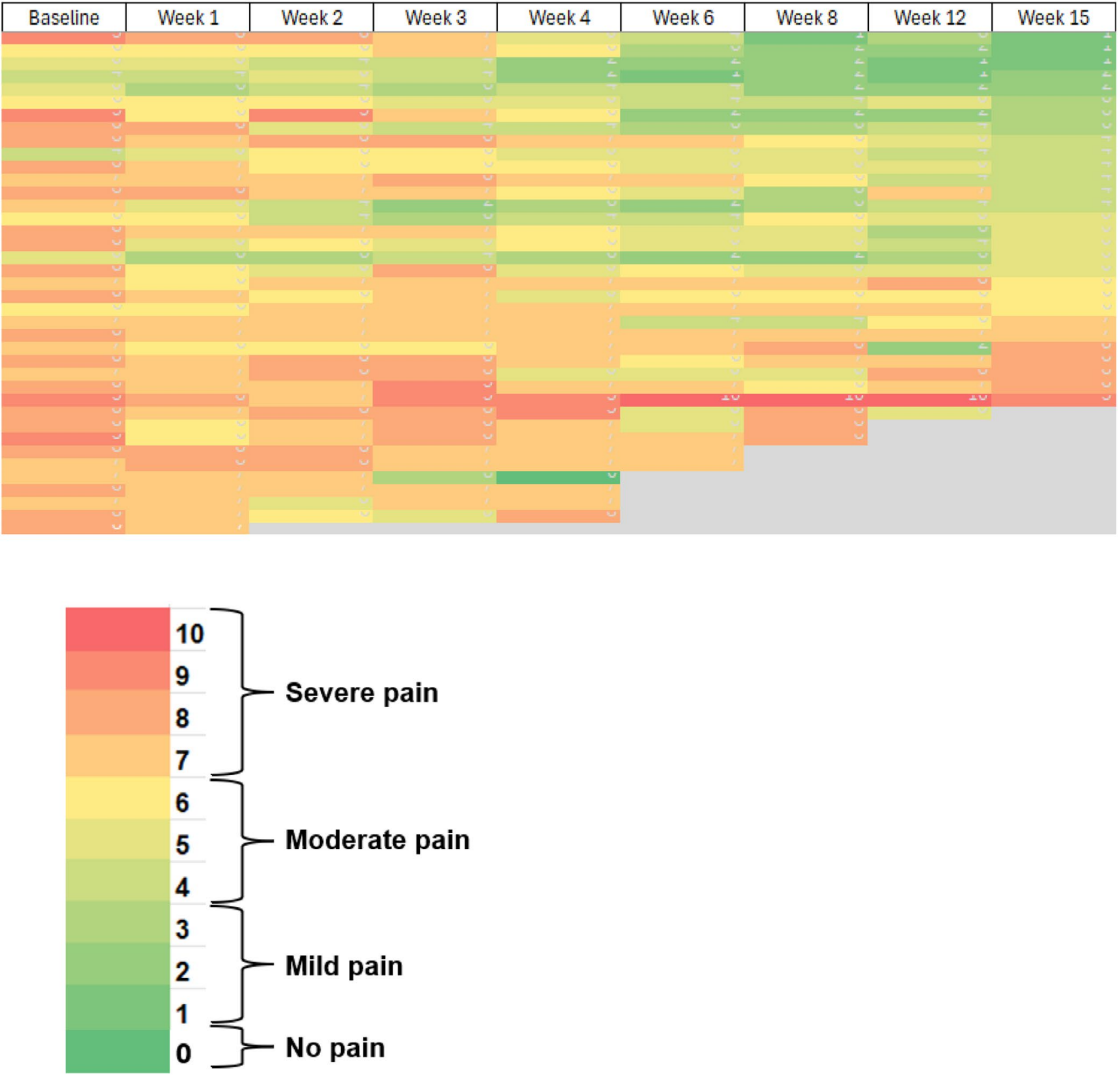
Heat map corresponding to change in worst pain for each participant during the study period. The X‐axis represents the time during the study while the y‐axis represents each study participant. Each lattice color represents a worst pain score on the Brief Pain Inventory (ranges from 0 to 10), with red representing more pain and green representing less pain. A change from red to yellow to green color represents an improvement in worst pain during the study period.

Based on the conservative assumption that the participants who did not undergo pain assessment at Week 15 did not have at least a 2‐point reduction in BPI worst pain, 17 of 39 participants (43.6%, 95% CI [28%, 60%]) achieved the pre‐defined primary outcome. In a sensitivity analysis including just the participants who completed the Week 15 assessment, 17 of 28 (61%) participants reported at least a 2‐point reduction (95% exact CI [0.41, 0.78]). Using a linear mixed model including data for all 39 participants, a higher baseline worst pain score was associated with higher subsequent worst pain scores (*p* < 0.001). For each week of treatment, BPI worst pain decreased by 0.13, leading to an average reduction of worst pain of 1.95 at the end of the 15‐week treatment period (Figure 2). BPI average pain decreased by 0.11 points per week, leading to a reduction of average pain of 1.65 points for evaluable participants over 15 weeks (*p* < 0.001). Similarly, the change in BPI pain interference with 15 weeks of CBD was 1.05 (*p* = 0.002).

When only data from the 28 participants who completed 15 weeks of treatment with CBD were included in the analysis, there was a clinically significant reduction in worst pain between baseline and Week 15 (average reduction of 2.36 points, 95% CI [−3.22, −1.49]). For average pain, 14 participants (50%) had at least a 2‐point reduction (95% CI 0.31–0.69). Finally, on the GRC scale, participants who completed treatment reported an average score of 2.43 (SD 1.37) for joint pain and 2.43 (SD 1.35) for joint stiffness.

### 3.4 Patient‐Reported Non‐Pain Outcomes

**FIGURE 3:**
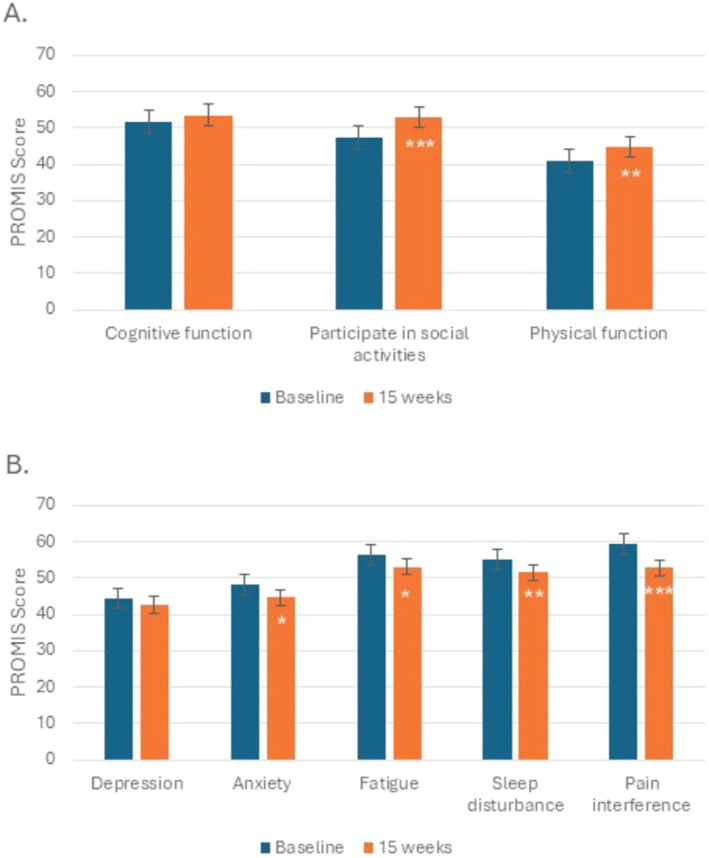
Change in PROMIS Outcome Measures for patients who completed cannabidiol therapy (*n* = 28). T‐scores are represented on the y‐axis, while PROMIS measure is on the x‐axis. An increase from baseline for a positively worded concept (A) reflects improvement, whereas an increase from baseline for a negatively worded concept (B) reflects worsening. The sign test was used because the differences do not follow the distributional assumptions of the *t* test. Asterisks represent statistical significance (*p* < 0.05*, *p* < 0.01**, *p* < 0.001***).

The effect of 15 weeks of CBD on other symptoms was explored. Response to the PROMIS questionnaires was unavailable for 1 participant. Using a linear mixed model, between Week 4 and Week 15 for all evaluable participants (*n* = 38), there was an improvement in ability to participate in social activities (2.71 points, *p* = 0.02), physical function (2.34 points, *p* = 0.01), and pain intensity (−0.68 points, *p* = 0.02). In the analysis that included only the participants who completed 15 weeks of CBD (*n* = 28), there was an improvement in ability to participate in social activities, anxiety, physical function, fatigue, sleep disturbance, and pain interference (Figure 3). For participants who completed 15 weeks of CBD (*n* = 28), there were improvements between baseline and Week 15 for the Fibromyalgia Survey (median 13.0 [range 1.00–26.0] vs. 8.50 [1.00–22.0], *p* < 0.001), PANAS Positive Scale (median 30.5 [range 18.0–44.0] vs. 38.9 [17.0–49.0], *p* < 0.001), and PANAS Negative Scale (median 14.5 [range 10.0–22.0] vs. 12.0 [10.0–25.0], *p* < 0.001). No difference was noted in catastrophizing over time.

### 3.5 Predictors of Change in Pain

To examine potential predictors of response to CBD, baseline PROs between responders and non‐responders were compared. PROMIS anxiety differed between these two groups; non‐responders had higher baseline levels of anxiety than responders (median 56.0 versus 40.3, *p* = 0.006). Using a linear mixed model, anxiety was also associated with worst pain, such that for each 1‐point increase in baseline anxiety scores, worst pain during the study period increased by 0.04 points (*p* = 0.02). There was no difference in baseline expectancy of response to CBD therapy between responders and non‐responders (median 12.0 [9.0–20.0] versus 13.0 [range 7.0–20.0], *p* = 0.86).

Using Spearman correlation, both baseline cognitive function and baseline anxiety were correlated with the change in BPI worst pain from baseline to Week 15. Those with higher baseline cognitive function scores had a larger decrease in worst pain (correlation = −0.42, *p* = 0.03), while those with higher baseline anxiety had a smaller decrease in worst pain (correlation = 0.41, *p* = 0.03). No significant correlations were seen for other baseline PROs and change in worst pain during the study period.

### 3.6 Change in Inflammatory Markers With CBD

A total of 38 participants had cytokine data at the baseline timepoint (at least one plasma, LPS, or null measurement). The number of participants with available cytokine data decreased by Week 8 (*n* = 27) and Week 15 (*n* = 20). For those with available unstimulated plasma samples, the median concentrations of the following cytokines decreased between baseline and Week 15 (*p* < 0.05): IL‐1β, IL‐13, IL‐15, IL‐17A, IL‐18, IL‐25, TNFα, MCP‐1, and IFNγ (Table S3). There was no change in stimulated cytokine concentrations between baseline and Week 15.

There were no associations identified between baseline unstimulated cytokine concentrations and response to CBD at Week 15. Responders to CBD therapy were more likely to have lower concentrations of unstimulated IL‐8 at Week 15 compared to non‐responders. No other associations between cytokine concentrations at Week 15 and response to CBD were found. In terms of change in pain and change in unstimulated cytokine concentrations over time, there were moderate positive correlations identified for IL‐7 and IL‐25, though neither reached statistical significance (*p* > 0.05); as each cytokine concentration decreased, worst pain decreased as well (Figure S3).

## 4 Discussion

While AIs are a key treatment for postmenopausal women with hormone receptor‐positive early‐stage breast cancer, the development of joint pain and muscle stiffness limits patients' quality of life and compliance with therapy, highlighting the need for effective treatment strategies [1, 3, 7, 31, 32]. Patients with breast cancer already report the use of over‐the‐counter CBD for the management of pain conditions, including joint stiffness, underscoring the need for formally testing a regulated CBD product in this population [33]. In this pilot clinical trial, the use of CBD for the treatment of postmenopausal women with AIMSS did not meet the pre‐defined primary endpoint of a 2‐point reduction in BPI worst pain. However, for patients who completed 15 weeks of CBD therapy, there was a clinically meaningful reduction in worst pain. While only looking at patients who completed treatment gives a potentially biased interpretation of the results, since half of those who stopped treatment early reported a lack of benefit, a subset of patients in this study clearly derived benefit from CBD.

Since pain‐related symptoms such as anxiety and insomnia are often reported by AI‐treated breast cancer survivors, and patients report taking CBD to improve sleep, this study also explored the impact of CBD on non‐pain outcomes [34]. For all participants, we found no difference in anxiety or insomnia with study therapy. Although there were statistically significant changes in self‐reported physical functioning, ability to participate in social activities, and pain intensity, based on prior research, the differences in these measures likely do not reflect a clinically meaningful improvement [35, 36]. However, the one‐point reduction in pain interference on the BPI has been suggested by IMMPACT guidelines to represent a minimally clinically important difference, defined as the smallest improvement considered worthwhile by a patient [37, 38, 39]. This suggests that CBD may have a clinically meaningful benefit in terms of pain interference reduction for this population. Similar to worst pain, a larger benefit in non‐pain outcomes was seen for participants who completed all 15 weeks of CBD therapy. In this group of participants, a lower degree of pain centralization and more positive affect were also reported at the end of the study period, suggesting additional patient characteristics that may be impacted by treatment with CBD.

Understanding which patients obtain benefit from treatment, and factors that predict response, will be important for the design of future studies of cannabinoids for treatment of AIMSS and for guiding personalization of therapy. Prior research suggests that patients with chronic pain may respond differently to different classes of analgesics based on the degree of pain centralization, such that individuals with higher degrees of central pain may be less likely to respond to anti‐inflammatory based treatment [40, 41]. More positive affect, lower degrees of catastrophizing, and high expectancy of benefit from treatment have also been associated with response to treatment [42, 43, 44, 45]. In this pilot study, many of the participants met diagnostic criteria for fibromyalgia at baseline and reported a high expectation that CBD would improve joint pain. However, there was no correlation between baseline measures of pain centralization, affect, catastrophizing, or expectancy of treatment and response to treatment with CBD. Alternatively, baseline anxiety was predictive, with higher baseline anxiety associated with less improvement in worst pain. This finding is consistent with other studies of the impact of baseline anxiety on change in chronic pain conditions [46, 47, 48, 49]. Additional research is needed to explore this relationship and whether screening for baseline anxiety and, if identified, subsequent treatment would be beneficial in the care of individuals with AIMSS.

Another important finding in this study is the safety of CBD use in patients with hormone receptor‐positive breast cancer. Marijuana has been shown to disrupt the ovulatory cycle and hormonal secretion, raising concern that it could impact estrogen levels, though it was unknown if CBD could have the same impact [50]. The efficacy of AI therapy in preventing breast cancer recurrence relies on the adequate suppression of estrogen production [31]. Findings from our pilot study demonstrate reassuring safety data, with no evidence of CBD causing increased estradiol concentrations using an ultrasensitive assay in AI‐treated patients.

Given that ongoing inflammation has been postulated to contribute to the development of AIMSS, this study also sought to evaluate the impact of CBD on circulating blood inflammatory markers [3, 34, 51]. In addition, research in individuals with chronic pain has found that an elevated stimulated immune response is common, suggesting these patients may have circulating immune cells that are primed to be pro‐inflammatory [52, 53, 54, 55]. Based on this data, both stimulated and unstimulated samples were analyzed in this pilot study. Interpretation is limited because of the relative lack of samples available from the later timepoints, which can impact the reliability of conclusions. While there was no difference in cytokine concentrations for stimulated samples between baseline and week 15, the concentration of multiple unstimulated cytokines decreased from baseline. Most cytokines that decreased were pro‐inflammatory, consistent with the known anti‐inflammatory properties of CBD [11, 56]. Interestingly, both IL‐7 and IL‐25, which have both pro‐ and anti‐inflammatory properties, were the only cytokines that had a moderate correlation with change in worst pain. The association of unstimulated IL‐8 concentration with response to therapy with CBD after 15 weeks also raises the question of whether lower levels of systemic inflammation could be associated with less AIMSS‐related pain. These associations warrant further preclinical investigation and replication in an independent cohort.

This was a rigorously conducted novel study in which a regulated form of CBD was evaluated in a population in need of treatment for AIMSS. However, there are limitations that should be considered when interpreting the results. First, this was a single‐center study with a largely homogenous population. The lack of diversity in demographics, including race/ethnicity and socioeconomic status, along with the relatively small sample size, limits the ability to draw strong conclusions from subgroup analyses or generalize the findings to a larger population. In future studies, it will be important to enroll a more representative population. While 39 patients were enrolled in this study, 11 patients discontinued study treatment prior to Week 15. For the 5 patients who discontinued CBD due to side effects (Table 3), we are unable to determine how their clinical response to CBD may have additionally impacted their decision to discontinue study treatment. As such, though not all participants discontinued treatment due to lack of response, the high treatment discontinuation rate and amount of missing data introduce potential bias in interpretation of the findings for participants who completed the treatment course. This potentially limits the ability to detect a true treatment effect and additionally impacts the generalizability of the results. Finally, while adjustments were not made for multiple comparisons in this exploratory pilot study, it will be important for future research with a larger sample size to include such comparisons.

As noted in other studies looking at the treatment of symptoms such as pain and hot flashes and in the absence of a control group, there is a substantial risk of placebo effect, which could impact the interpretation of the results [57, 58]. Finally, while the dose of CBD was chosen to limit side effects while maintaining the potential for analgesic benefit, it is possible that the dose studied in this clinical trial was too low to see the maximum benefit in patients with AIMSS [59, 60, 61]. CBD and anastrozole serum concentrations were not evaluated as part of this pilot study, as the focus was on the safety of CBD use in regard to its impact on circulating estradiol, along with markers of inflammation. However, future studies should include biomarkers to assess whether there are relationships between serum concentration of CBD and health outcomes, both patient‐reported and objective measures. In addition, given the inhibition of CYP3A4 by CBD and potential reduction in serum concentrations of anastrozole, future clinical trials studying the combination of CBD and AI therapy should examine this possibility in more detail [62]. This could also be clinically relevant for patients who are taking over‐the‐counter CBD while on AI treatment for breast cancer.

## 5 Conclusion

Treatment with CBD was associated with an improvement in AIMSS for a subset of patients. Use of CBD was safe and tolerable for women with hormone receptor‐positive breast cancer. Associations were seen between baseline anxiety and worst pain, as well as cytokine concentration and treatment response. These findings suggest the need for additional studies to further understand the etiology of AIMSS, and to explore which patients are most likely to benefit from treatment with CBD.

## Author Contributions

**Nicole M. G. Fleege:** conceptualization (equal), funding acquisition (equal), visualization (equal), writing – original draft (equal), writing – review and editing (equal). **Elise A. Miller:** formal analysis (equal), visualization (equal), writing – review and editing (equal). **Kelley M. Kidwell:** formal analysis (equal), visualization (equal), writing – review and editing (equal). **Zeb R. Zacharias:** investigation (equal), resources (equal), writing – review and editing (equal). **Jon Houtman:** investigation (equal), resources (equal). **Kelly Scheu:** resources (equal), writing – review and editing (equal). **Kathleen Kemmer:** resources (equal), writing – review and editing (equal). **Kevin F. Boehnke:** conceptualization (equal), writing – review and editing (equal). **N. Lynn Henry:** conceptualization (equal), funding acquisition (equal), resources (equal), supervision (equal), visualization (equal), writing – original draft (equal), writing – review and editing (equal).

## Ethics Statement

The study was approved by the University of Michigan Institutional Review Board, and all participants provided written informed consent prior to enrollment.

## Conflicts of Interest

N.L.H. is a local PI for a clinical trial sponsored by Blue Note Therapeutics, serves as a consultant for Myovant Pharmaceuticals and AstraZeneca, and receives royalties from Up‐to‐Date, none of which are related to the research presented in this manuscript. K.F.B. has received grant funding from Tryp Therapeutics for a clinical trial of psilocybin‐assisted therapy and sits on a data safety and monitoring board for an ongoing clinical trial with Vireo Health (unpaid). He also received grant funding for protocol development from Journey Biosciences. He has received grant funding from the National Institute on Drug Abuse and the National Institutes of Arthritis, Musculoskeletal, and Skin Diseases of the National Institutes of Health. He has also received grant funding from the State of Michigan Veteran Marijuana Research Program. K.F.B. has received speaking fees for lectures from the Medical Cannabis Research Advocacy Alliance, Provide Holy Cross Medical Center, the University of Michigan Retirees Association, and the Michigan Center of Clinical Systems Improvement, as well as an honorarium from Viatris Inc. for a podcast on fibromyalgia through European Medical Journal.

## Supporting information


**Data S1:** cam471117‐sup‐0001‐Supinfo.docx.

## Data Availability

The data that support the findings of this study are available on request from the corresponding author. The data are not publicly available due to privacy or ethical restrictions.
