# Supplementary material for: Pilot Study of Cannabidiol for Treatment of Aromatase Inhibitor‐Associated Musculoskeletal Symptoms in Breast Cancer
Source: Cancer Med. 2025 Aug 1;14(15):e71117. doi: 10.1002/cam4.71117 (PMC12316815; doi:10.1002/cam4.71117)
Supplement: Supplementary file 1 — Data S1: cam471117‐sup‐0001‐Supinfo.docx. [file CAM4-14-e71117-s001.docx]

**Supplemental Tables and Figures**

**Supplemental Table 1. Inclusion and Exclusion Criteria.** LHRH = luteinizing hormone-releasing hormone; AI = aromatase inhibitor; HER2 = human epidermal growth factor receptor 2; CDK = cyclin-dependent kinases; ALT = alanine transaminase; AST = aspartate aminotransferase; ULN = upper limit normal; THC = tetrahydrocannabinol; CBD = cannabidiol.

| **Inclusion Criteria** | **Exclusion Criteria** |
| --- | --- |
| - Female subject aged ≥ 18 years who are postmenopausal according to standard clinical criteria (absence of menses for at least 12 months, prior bilateral salpingo-oophorectomy, serum or plasma concentrations of estradiol within postmenopausal range according to local lab values) or who will have been receiving concomitant LHRH agonist therapy. - Taking aromatase inhibitor therapy (anastrozole, exemestane, or letrozole) for adjuvant treatment of breast cancer or for chemoprevention for at least 3 weeks and no more than 2 years at the time of enrollment. - Planning to take the same AI therapy for at least 15 weeks. - New or worsening joint pain since starting the AI therapy, with worst pain score of at least 4 out of 10 over the 7 days prior to enrollment. - Completion of surgery (mastectomy or lumpectomy/partial mastectomy) for treatment of breast cancer at least 3 months prior to enrollment. Completion of axillary surgery as indicated (not required). - Completion of chemotherapy, if given. Concurrent use of LHRH agonist therapy, anti-HER2 therapy, bisphosphonate therapy, and CDK4/6 inhibitor therapy is permitted. - Patients receiving treatment with opioids, duloxetine, gabapentin, and/or pregabalin must have been taking a stable dose for at least 30 days prior to enrollment. - ECOG Performance Status 0-2. - Able to self-complete questionnaires in English. - Able to provide informed consent and willing to sign an approved consent form that conforms to federal and institutional guidelines. | - Metastatic breast cancer. - Planned surgery during the 8-week study period. - ALT/AST > 3x ULN or bilirubin > 1.5x ULN. If known Gilbert’s syndrome, bilirubin may not be > 2x ULN. - History of suicide attempt. - History of seizure other than febrile seizures in childhood. - Current use of valproate or clobazam. If patient takes valproate or clobazam, should discontinue 7 days prior to enrollment. - Use of cannabidiol, THC, or marijuana (oral, inhaled, or topical) within the 6 weeks prior to enrollment. - Known allergy or hypersensitivity to CBD or any of the excipients in the medication, including sesame. - Unable to take oral medications. - Pregnant or breast feeding. - Any medical condition that would interfere with the absorption of study medication. Prior gastric bypass is permitted. - Concurrent medical or arthritis disease such as active rheumatoid arthritis or inflammatory arthritis that could confound or interfere with evaluation of pain or efficacy. Patients with osteoarthritis are eligible. - Patients with a prior or concurrent malignancy whose natural history or treatment, in the opinion of the treating investigator, has the potential to interfere with the safety or efficacy assessment of the investigational regimen. |

**Supplemental Table 2. Description of Validated Questionnaires Collected During the Study Period.**

| **Outcome** | **Measure** |
| --- | --- |
| Brief Pain Inventory-Short Form (BPI) | A validated 14-item pain assessment tool to assess pain and symptom burden. It uses a numeric rating scale from 0-10 to rate pain from mild to severe. This scale has been validated for use in patients with cancer pain (reference 25) and has been studied in patients with AIMSS^5,6,18^. |
| PROMIS Profile 29+2 v2.1 | A validated 26-item questionnaire that assesses patient-reported symptoms over the past 7 days in 9 PROMIS domains (fatigue, sleep disturbance, physical function, depression, anxiety, ability to participate in social roles and activities, and cognitive function). Raw scores for each domain are calculated and converted to a T-score, with a mean of 50 and standard deviation of 10. Higher T scores represent more of the concept being measured^21^. |
| Global Ratings of Change (GRC) Scale | A single-item assessment tool that assesses how a condition has improved, worsened, or stayed the same compared to a previous point in time^22^. Change is rated on a 7-point Likert scale that ranges from -3 (“a very great deal worse”) to +3 (“a very great deal better”). |
| 2011 Fibromyalgia Survey | A combined measure of widespread pain (body map of painful sites) and symptom severity (i.e. fatigue, cognitive problems, headache, poor mood), as a self-reported proxy of centralized pain^24^. A diagnosis of fibromyalgia is made when symptoms have been present for at least 3 months and when widespread pain index (WPI) is at least 7 and symptom severity score (SS) is at least 5 or when WPI is between 3-6 and SS is at least 9. |
| Coping Strategies Questionnaire Catastrophizing Scale | A validated 6-item subscale that assesses pain catastrophizing, a thinking style that has been associated with the progression of acute pain to chronic states, and with poorer outcomes generally^25^. Each item is scored from 0 (“never do that”) to 6 (“always do that”) |
| Positive and Negative Affect Scale (PANAS) | A validated 20-item instrument that measures positive and negative affect^26^. It can be scored to include a metric of affect balance (i.e. greater negativity over positivity)^60^. Each question is rated on a 5-point Likert scale, where 1 means “very slightly or not at all” and 5 means “extremely.” Scores for both positive and negative affect questions can range from 10 to 50. The higher the score on the positive affect questions, the higher levels of positive affect. Lower scores on the negative affect questions indicated lower levels of negative affect. |
| Mao Expectancy of Treatment Effects (METE) | A validated 4-item self-report questionnaire rated on a scale of 1-5 (from total disagreement to total agreement), assessing a patient’s expectation that CBD will relieve AIMSS symptoms^43^. Total score can range from 4-20, with a higher score indicating greater expectation that CBD will improve joint pain. This has good internal consistency in a prior study of acupuncture in patients with cancer^23^. |

**Supplemental Table 3. Comparing Plasma Cytokine Values between Baseline and Week 15.** All values listed as mean (standard deviation) unless otherwise specified. P-values were calculated using the Wilcoxon Signed-Rank test. IL = interleukin, MCP = monocyte chemoattractant protein, TNF = tumor necrosis factor, IFN = interferon.

| **Cytokine** | **Baseline (n = 19)** | **Week 15 (n = 19)** | **p-value** |
| --- | --- | --- | --- |
| IL-1b | 150 (565) | 15.0 (25.0) | 0.0027 |
| IL-13 | 662 (1710) | 147 (218) | 0.0015 |
| IL-15 | 32.6 (82.4) | 10.1 (7.64) | 0.047 |
| IL-17A | 88.1 (248) | 15.0 (15.1) | 0.018 |
| IL-18 | 121 (284) | 35.2 (22.3) | 0.026 |
| IL-25 | 15300 (50200) | 2820 (5250) | < 0.001 |
| MCP-1 | 330 (377) | 217 (67.6) | 0.04 |
| TNF-alpha | 188 (635) | 26.0 (34.4) | 0.0017 |
| IFN-gamma | 155 (319) | 54.9 (69.9) | 0.026 |

**Supplemental Table 4. Reasons for Study Withdrawal.** A description of the reasons for study withdrawal prior to trial completion (n = 11).

| **Patient** | **Reason** | **Details** |
| --- | --- | --- |
| 1 | Patient discretion | Felt to be ineffective |
| 2 | Patient discretion | Felt to be ineffective |
| 3 | Patient discretion | Felt to be ineffective |
| 4 | Patient discretion | Felt to be ineffective |
| 5 | Patient discretion | Felt to be ineffective |
| 6 | Patient discretion | Worsening of AIMSS symptoms |
| 7 | Adverse event | Grade 1 fatigue |
| 8 | Adverse event | Grade 2 neuropathy |
| 9 | Adverse event | Grade 2 rash |
| 10 | Adverse event | Grade 2 agitation |
| 11 | Adverse event | Grade 1 dizziness, grade 1 stomach pain |

**Supplemental Figure 1. Study Calendar**

**
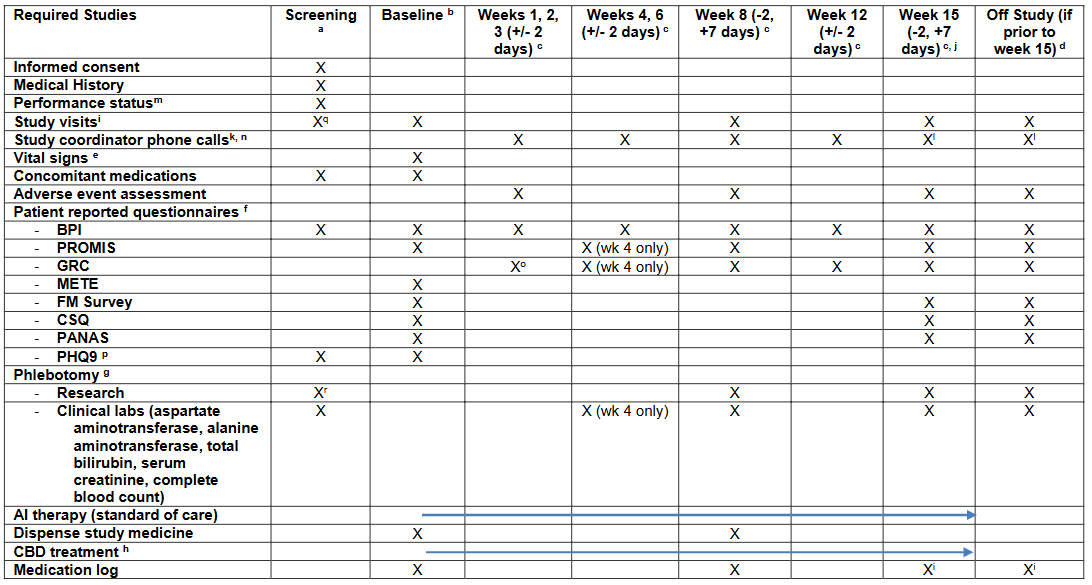
**

**Supplemental Figure 2. Missing data.** Data should have been collected at 10 time points between baseline and week 15 for the 39 patients (= 390 time points). Across all participants, data were not collected at 46 time points (missing 11.8% of data overall). For participants who completed the study (n = 28, 280 expected time points), data were missing for only 10 time points (3.6%).


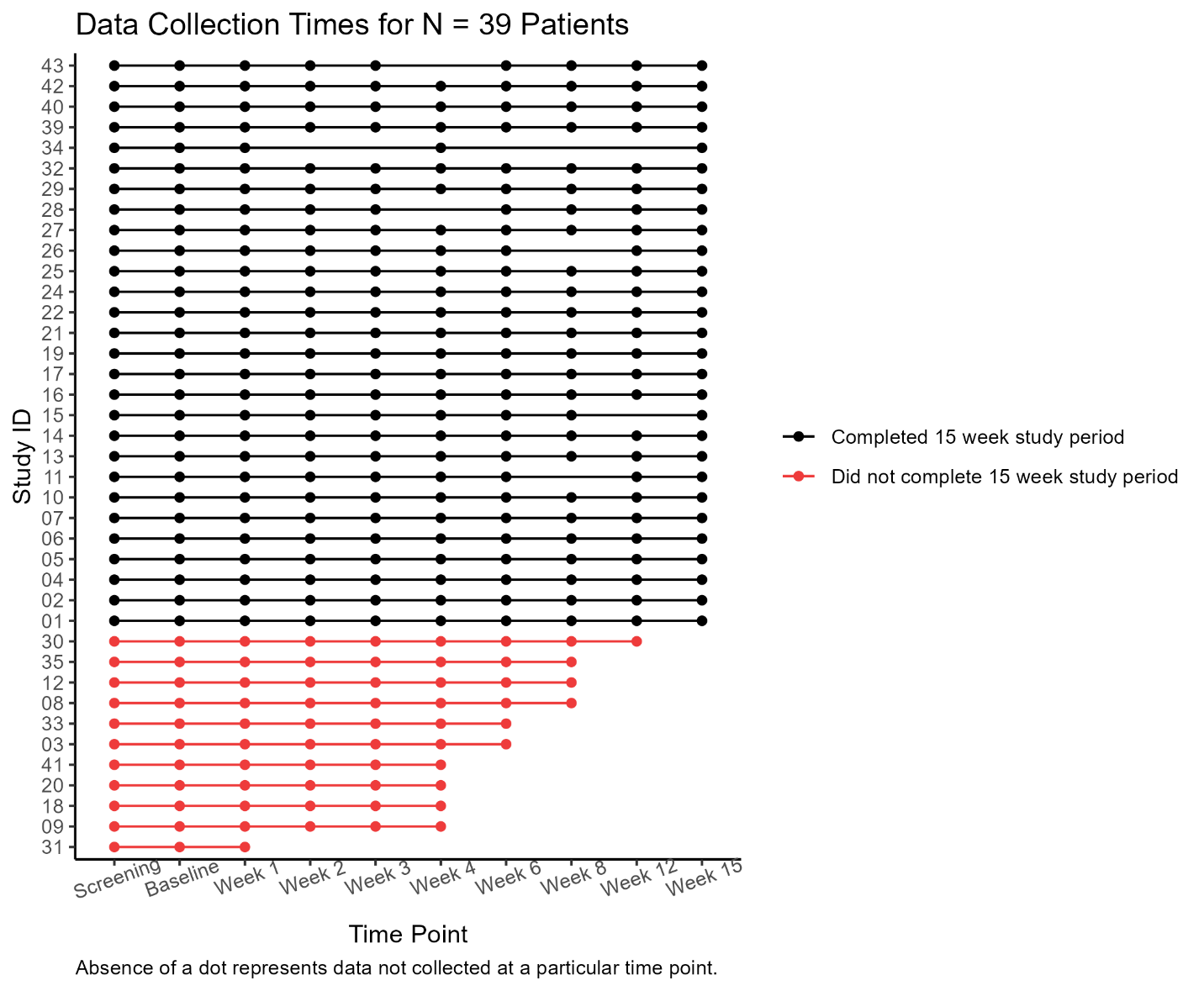


**Supplemental Figure 3. Correlation Between Difference in Unstimulated Cytokine Concentration from Baseline to Week 15 and Difference in Worst Pain from Baseline to Week 15.** Y-axis represents the change in worst pain while X-axis represents the change in cytokine concentration for (A) unstimulated IL-7 and (B) unstimulated IL-25. R is the Spearman Correlation. Dotted red lines are drawn where the difference is 0.

A.

**
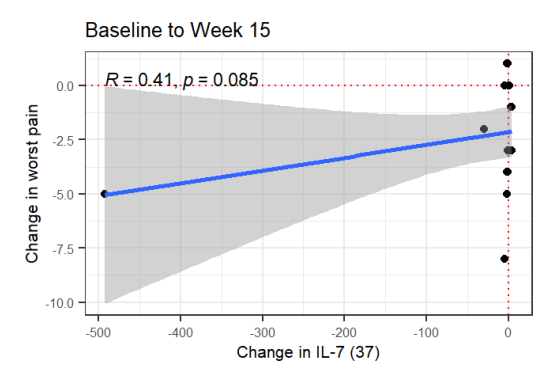
**

B.

**
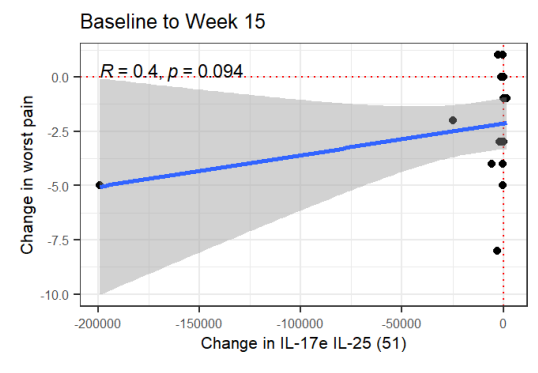
**
